# 급성 심부전 응급실 환자의 고유량 비강 캐뉼라 치료 실패 예측 모델 개발: 후향적 이차 자료 분석

**DOI:** 10.7586/jkbns.26.030

**Published:** 2026-08-25

**Authors:** Sun-Ja Kim, Sun Hyoung Bae

**Affiliations:** 1 College of Nursing, Ajou University, Suwon, Korea; 1 아주대학교 간호대학; 2 Department of Nursing, Hallym University Kangnam Sacred Heart Hospital, Seoul, Korea; 2 한림대학교 강남성심병원 간호부; 3 Research Institute of Nursing Science, College of Nursing, Ajou University, Suwon, Korea; 3 아주대학교 간호대학 간호과학연구소

**Keywords:** Oxygen inhalation therapy, Heart failure, Machine learning, Emergency nursing, Decision support systems, clinical, 산소흡입요법, 심부전, 머신러닝, 응급간호, 임상의사결정지원시스템

## Abstract

**Purpose:**

This study developed and internally validated a model integrating oxygenation, acid–base, and severity variables for high-flow nasal cannula (HFNC) failure in emergency department (ED) patients with acute heart failure (AHF).

**Methods:**

This retrospective secondary analysis included adults with AHF who received HFNC therapy in a tertiary ED between January 2021 and August 2025. Five prespecified predictors—follow-up saturation-to-fraction of inspired oxygen (S/F) ratio, follow-up respiratory rate–oxygenation index incorporating heart rate, norepinephrine use, Sequential Organ Failure Assessment score, and arterial pH—were entered into logistic regression and four machine learning algorithms using R 4.3.5 program.

**Results:**

Among 201 patients, 33 (16.4%) experienced HFNC failure. In the held-out test set, the five-variable logistic regression model showed an area under the receiver operating characteristic curve (AUROC) of .70 (95% confidence interval [CI], .51–.88), with a bootstrap optimism-corrected AUROC of .78 (95% CI, .70–.86) in the full cohort. The five models showed AUROCs ranging from .70 to .81, with overlapping confidence intervals. Per 1-standard deviation increase, the follow-up S/F ratio (odds ratio [OR], 0.20; 95% CI, 0.07–0.58) and arterial pH (OR, 0.55; 95% CI, 0.35–0.86) were independent predictors of HFNC failure, whereas norepinephrine use was not (OR, 2.70; 95% CI, 0.37–19.50).

**Conclusion:**

Integrating early oxygenation response with acid–base status may assist in the bedside stratification of HFNC failure risk in AHF. Standard logistic regression using these prespecified parameters may offer an interpretable alternative to machine learning models, although it did not outperform the follow-up S/F ratio alone.

## 서론

급성 심부전(acute heart failure, AHF)은 심장 펌프 기능의 급격한 악화로 혈역학적 불안정과 폐울혈을 초래하는 생명을 위협하는 임상 증후군이다[1,2]. AHF에서 산소화 장애는 일반적으로 폐부종과 순환 부전이 복합된 맥락에서 발생하므로, 환자 평가 시 산소화 지표와 함께 혈압, 말초 관류, 호흡 역학과 같은 순환 및 환기 지표를 통합적으로 고려해야 한다[3]. 호흡곤란, 의식 변화, 혈역학적 불안정은 수 분에서 수 시간 내에 진행될 수 있어 이러한 환자는 흔히 응급실에 내원하며, 응급실에서는 신속한 평가와 안정화가 요구된다[4,5]. 응급실은 중환자실과 달리 환자의 중증도가 다양하고 간호 인력이 제한적이며 다수의 중증 환자를 동시에 관리해야 하는 특성을 가진다[6]. 이러한 환경에서 간호사는 산소포화도 (SpO_2_), 호흡수, 혈압, 심박수, 말초 관류, 의식 수준 (Glasgow Coma Scale, GCS)을 종합하여 시간에 민감한 임상 의사결정을 내려야 한다[7,8].

고유량 비강 캐뉼라 (high-flow nasal cannula, HFNC) 요법은 비강 인터페이스를 통해 가온·가습 된 가스를 고유량으로 전달하여 유량 의존적 저수준 양압 (flow-dependent low level positive airway pressure), 안정적인 흡입산소분율 (fraction of inspired oxygen, FiO_2_), 사강 제거 (dead-space washout)를 제공한다[9]. 이러한 기전을 통해 HFNC는 호흡곤란을 완화하고 산소화를 개선하며, 응급실에서 일차 산소화 전략으로 그 사용이 확대되어 왔다[10,11]. 심인성 폐부종 및 AHF 환자에서 HFNC는 호흡수 감소와 초기 산소화 개선 등 기존 산소요법보다 우수한 생리적 이점을 보인다[10,11]. 그러나 HFNC가 모든 환자에서 동일하게 효과적이지는 않으며, 실패 위험이 높은 환자에서 HFNC를 지속하면 기관내 삽관이 지연될 수 있고, 침습적 환기 이전의 장시간 HFNC 적용은 원내 사망률 증가와 독립적으로 연관된다[12-14]. 따라서 치료 실패를 조기에 인지하고 적시에 호흡 보조를 강화하는 것이 필요하다[13,14].

HFNC 반응을 조기에 평가하기 위해 산소화와 호흡 부하를 함께 반영하는 지표가 활용되며, 대표적으로 호흡수-산소화 (respiratory rate–oxygenation, ROX) 지수가 있다[15,16]. 최근 여러 연구에서는 활력징후, 산소화 지표, 동맥혈가스 등 다양한 임상 변수를 통합한 머신러닝 (machine learning) 기반 모형을 기관내 삽관과 임상 악화 예측에 적용하고 있다[17,18]. 그러나 이러한 모형은 대부분 폐렴과 급성호흡곤란증후군이 주를 이루는 비심인성 급성 저산소성 호흡부전 코호트에서 산소화 지표를 중심으로 개발되었으며, ROX 지수나 산소포화도 대 흡입산소분율 비 (saturation to fraction of inspired oxygen ratio, S/F ratio)와 같은 산소화 중심 지표는 산소화와 호흡 상태를 평가하는 데 유용하지만, AHF의 임상 경과를 좌우하는 환기·산–염기 상태와 혈역학적 불안정, 전신 장기 기능부전을 반영하지 못한다는 한계가 있다[2,3,10,19,20]. 특히 AHF에서 폐포 부종과 호흡근 피로가 진행하면 고탄산혈증성 환기부전이 동반될 수 있으나, SpO_2_와 FiO_2_만으로 산출되는 S/F ratio는 이러한 환기 상태를 포착하지 못한다[3,19].

HFNC 실패를 조기에 인지하는 것은 적시의 호흡 보조 강화로 이어져 삽관 지연에 따른 예후 악화를 줄일 수 있다는 점에서 임상적으로 중요하며[12-14], 이를 위해서는 응급실에서 위험을 신속히 선별할 수 있는 신뢰성 있는 예측도구가 요구된다. 머신러닝 기반 모형은 산소화·혈역학·전신 중증도 등 서로 다른 영역의 변수를 함께 다룰 수 있어, 산소화 중심 지표만으로는 포착하기 어려운 AHF의 위험 요인을 반영하는 데 적합하다[17,19]. 또한 HFNC 적용 직후의 초기 치료 반응은 이후 임상 경과를 예측하는 데 유용한 정보이므로[15,16], 이러한 초기 반응을 내원 시점의 임상 상태와 함께 평가하면 단일 시점 평가보다 더 정확한 위험 평가가 가능하다. 그러나 AHF 환자를 대상으로 산소화·혈역학·전신 중증도 변수를 통합한 머신러닝 기반 HFNC 치료 실패 예측모형 개발 연구는 미흡한 실정으로, 응급실 임상 현장에서 활용 가능한 예측도구 개발이 필요하다. 이에 본 연구는 응급실 AHF 환자에서 HFNC 적용 전 (기저 시점)과 HFNC 적용 후 30분–2시간 (추적 시점)의 생리·혈역학·임상 변수를 통합한 머신러닝 기반 HFNC 치료 실패 예측모형을 개발하고 내적으로 검증하며, 무작위 숲 (random forest, RF), 의사결정나무 (decision tree, DT), 방사기저함수 커널 서포트 벡터 머신 (support vector machine, SVM), 경사 부스팅 (gradient boosting, GB)의 네 가지 머신러닝 알고리즘의 식별 성능을 로지스틱 회귀 (logistic regression, LR) 기준 모형과 비교하고, HFNC 치료 실패와 연관된 핵심 임상 변수를 규명하기 위해 수행되었다. 본 연구를 통해 개발된 예측모형은 응급실 간호사가 HFNC 적용 환자의 치료 실패 위험을 조기에 선별하고 적시에 의료진에게 보고하는 임상적 판단을 지원하는 데 기여할 것으로 기대된다.

## 연구 방법

### 1. 연구 설계

본 연구는 응급실에 내원한 급성 심부전 환자에서 고유량 비강 캐뉼라 치료 실패를 예측하는 다변량 모형을 개발하고 내적 검증한 후향적 관찰 연구이다. 보고는 STROBE 지침[21]과 임상 예측모형 보고지침인 TRIPOD+AI [22]를 따랐다.

### 2. 연구 대상

2021년 1월부터 2025년 8월까지 서울 소재 일개 상급종합병원 응급의료센터에 내원한 만 18세 이상의 심부전 (ICD-10, I50.0~I50.9) 환자 중 HFNC 산소요법을 적용 받은 환자의 전자의무기록 (EMR)을 분석하였다. 연명의료 중단 및 심폐소생술 포기 (5명), 타원 전원 (2명), 자의 퇴원 (2명), 데이터 결측 (2명)에 해당하는 11명을 제외하고 최종 201명을 분석하였으며, 이 중 33명 (16.4%)이 HFNC 치료 실패로 분류되었다.

대상자의 표본 크기는 임상 예측모형의 표본 크기 기준[23]에 따라 평가하였다. 응급실 HFNC 실패 선행연구[15,16,24]에서 보고된 식별 성능, 즉 수신자 조작 특성 곡선하 면적 (area under the receiver operating characteristic curve, AUROC) .80~.90을 참조하여, 기대 AUROC를 .85로 가정하였을 때, Cox–Snell R2 = .22, 목표 축소계수 (shrinkage factor) .90 이상, apparent R²의 낙관도 (optimism) .05 이하의 기준을 모두 충족하여 본 연구의 표본 수는 적절하였다.

### 3. 연구변수

#### 1) 종속 변수

종속 변수는 HFNC 치료 실패로, HFNC 적용 후 응급실 체류 중 기관내 삽관 또는 침습적 기계환기로 전환된 경우로 정의하였다[13,16]. HFNC 적용과 삽관 전환의 결정은 응급의학과 전문의의 임상적 판단에 따라 본 의료기관 응급의료센터의 표준 진료에 준하여 이루어졌다. 모든 사건은 EMR에 기록된 임상 사건에 근거하여 두 명의 연구자가 독립적으로 추출하였고, 불일치 시 합의를 통해 이분형으로 코딩하였다.

#### 2) 독립 변수

독립 변수 후보로는 HFNC 치료 실패 예측에 관한 선행연구[3,13,15-17,19]와 급성 심부전 임상 진료지침[1]에 근거하여, EMR에서 추출 가능하고 응급실에서 측정할 수 있는 33개 변수를 선정하였다. 후보 변수는 인구사회학적 특성, 내원 시점 및 추적 시점 활력징후, 응급실 내 중재, 혈액화학 및 동맥혈가스 검사, 산소화 지수, 중증도 지표의 여섯 개 영역으로 구성되었다. 이 중 성별은 의무기록상 기재된 생물학적 성별 (sex)을 기준으로 분류하였고, 연령, GCS, 동맥혈가스 검사, 혈액화학 검사, Sequential Organ Failure Assessment (SOFA) 총점은 기저 시점에 측정하였다. 활력징후와 호흡-산소화 지표는 HFNC 적용 전 (기저 시점)과 적용 후 30분~2시간 이내 (추적 시점)에 측정하였다. 환자별로 단일 측정값을 확보하기 위해 추적 시간 창 내에서 2시간 시점에 가장 가까운 값을 채택하였다. 단, 해당 시간 창 내에 기관내 삽관을 시행한 환자는 삽관 직전에 마지막으로 측정된 값을 추적 시점의 값으로 정의하였다. 실제 추적 시점 측정은 HFNC 적용 후 중앙값 68.0분 (interquartile range [IQR] 53.0~86.0 분)에 이루어졌으며, 치료 실패군 (median 60.0분, IQR 50.0~89.0 분)과 성공군 (median 70.0분, IQR 53.0~85.2 분) 간 측정 시점의 차이는 통계적으로 유의하지 않았다 (Mann-Whitney U = 2929.0, *p* = .608). 산소포화도 대 흡입산소분율 비 (S/F ratio)와 ROX index는 기저 시점의 값과 추적 시점의 값을 구분하여 사용하였다. 특히 ROX 계열 지표는 HFNC 적용 후 측정한 값의 치료 실패 예측력이 더 우수하다는 선행연구[15,16,24]에 따라, 기저 시점의 값은 군간 특성 비교에만 사용하고 예측모형에는 추적 시점의 값을 입력하였다.

본 연구는 결과 사건 (event) 발생 건수가 제한적이므로, 모든 후보 변수를 예측모형에 투입할 경우 과적합과 모형의 불안정성을 초래할 위험이 높다[23,25]. 따라서 자료 기반 변수 선택 절차를 적용하는 대신 산소화·혈역학·전신 중증도·산–염기라는 서로 다른 생리적 영역을 각각 대표하도록 선행연구와 임상 진료지침에 근거하여 다섯 개 변수를 예측 변수로 사전 정의하였다[22,23,25]. 구체적으로 HFNC 치료 실패의 예측 지표로 보고된 추적 S/F ratio [16]와 추적 ROX-심박수 지수[15], 혈역학적 불안정을 반영하는 노르에피네프린 사용[1,13], 다장기 기능부전의 통합 지표인 SOFA 총점[17,26], 환기·산–염기 상태를 반영하는 동맥혈 pH [3,19]를 선정하였다. 아울러 후보 변수 33개 전체를 대상으로 표본의 절반을 무작위로 추출하는 과정을 1,000회 반복하는 LASSO 안정성 선택 (stability selection)을 수행하여 자료 기반의 변수 선택 경향을 확인하였다[27] (Supplementary 1 Part A). 단, 사건 수가 적은 자료에서는 이러한 자료 기반 선택이 불안정할 수 있으므로, 이를 임상적 근거로 사전 정의한 예측변수를 대체하는 대신 보조 분석으로만 활용하였다. 선정된 다섯 개 변수 간의 다중공선성은 분산팽창지수 (variance inflation factor) 5 미만을 기준으로 확인하였다 (Supplementary 1 Part B).

한편, 좌심실 박출률 (left ventricular ejection fraction)과 European Society of Cardiology 지침 기반 임상 표현형 (pulmonary edema-dominant, hypoperfusion-dominant, cardiogenic shock, right ventricular failure) 분류는 응급실 내원 당시의 EMR에 구조화된 형태로 기록되어 있지 않아 모형 입력에서 제외하였다.

### 4. 자료 수집

구조화된 EMR에서 표준화된 추출 절차에 따라 자료를 수집하였다. 연구 기간 동안 선정 기준을 충족하는 환자를 식별한 뒤 일반적 특성, 활력징후, 검사 결과, 치료 정보를 추출하였으며, 수집된 모든 자료는 분석 전 비식별화 과정을 거쳤다.

### 5. 자료 분석

대상자 특성은 연속 변수의 경우 정규성에 따라 평균과 표준편차 또는 중앙값과 사분위수 범위로, 범주형 변수의 경우 빈도와 백분율로 제시하였다. 모든 분석은 R 4.3.5 (R Foundation for Statistical Computing, Vienna, Austria)로 수행하였다.

#### 1) 자료 전처리

본 연구의 분석에 사용된 데이터 세트에는 결측치가 없었다. 데이터 전처리 과정에서 심한 비대칭 분포를 보이는 혈액 검사 지표 (B-type natriuretic peptide, creatinine, lactate, total bilirubin)에 대해서는 자연로그 변환을 수행하였다. 이상치 검토 과정에서 HFNC 적용 후 체온이 3,630으로 기재된 1건은 명백한 소수점 입력 오류로 간주하여 36.30으로 수정하였다. 아울러, 변수 간 척도 차이가 머신러닝 모형에 미치는 영향을 통제하기 위해, 모든 연속형 예측변수는 모형 적합 전 평균 0, 표준편차 1을 갖도록 표준화 (standardization)하여 분석에 사용하였다.

#### 2) 예측모형 개발

일차 모형으로 LR을, 비교 모형으로 RF, DT, SVM, GB 등 네 가지 머신러닝 알고리즘을 사용하여 예측모형을 구축하였다[17,19]. 또한 사건 수가 제한적인 점을 고려하여, 다섯 개 변수 모형과 함께 추적 시점 S/F ratio, 동맥혈 pH, SOFA 총점으로 구성한 간명 모형 (parsimonious model)을 민감도 분석의 일환으로 함께 구축하였다. 전체 자료는 결과 변수를 기준으로 층화 하여 학습 자료 70% (n = 141)와 검증 자료 30% (n = 60)로 분할하였으며, 검증 자료는 최종 성능 추정에만 사용하여 정보 누출 (information leakage)을 방지하였다[28]. 머신러닝 알고리즘의 하이퍼파라미터 (hyperparameter)는 학습 자료에 한정하여 5-겹 교차검증 (5-fold cross-validation) 기반 격자 탐색 (grid search)으로 최적화하였으며, 평균 AUROC가 가장 높은 조합을 최종 선택하였다. 각 알고리즘의 탐색 범위와 최종 선택 값은 다음과 같다. RF의 mtry는 1, 2, 3 중 1로, DT의 cp는 0.001~0.05 범위에서 0.05로 결정하였다. SVM은 비용 C를 0.1, 1, 10 중 0.1로, 커널 폭 σ를 0.01, 0.1, 1 중 0.01로 설정하였다. GB는 트리 수를 100, 300 중 100으로, 최대 깊이를 2, 3, 4 중 2로, 학습률을 0.05, 0.1 중 0.1로, min_child_weight를 5, 10 중 5로 각각 지정하였다.

#### 3) 내적 검증 및 클래스 불균형

일차 모형인 LR의 내적 타당도는 두 가지 방법으로 평가하였다. 첫 번째로, 전체 자료 (N = 201)에 1,000회 부트스트랩 낙관도 (optimism) 보정을 적용하여 낙관도 보정 AUROC와 95% 신뢰구간 (confidence interval, CI)을 산출하였다[29]. 두 번째로, 무작위 시드 (random seed)를 달리한 70:30 층화 분할을 50회 반복하여 AUROC의 95% 경험적 범위 (2.5~97.5백분위수)를 확인하였다.

단일 분할의 한계를 보완하기 위해 10-겹 교차검증 (10-fold cross-validation)을 20회 반복하여 다섯 개 변수 모형과 단일 추적 시점 S/F ratio 모형의 식별 성능, 정밀도-재현율 곡선하 면적 (area under the precision-recall curve, AUPRC), 보정 성능을 비교하였으며, 두 모형의 AUROC 차이에 대한 신뢰구간은 부트스트랩으로 추정하였다. 아울러 임상적 의사결정 기준이 되는 임계확률 전 범위에서 결정곡선분석 (decision curve analysis, DCA)을 시행하여 두 모형의 임상적 유용성을 비교하였다. 한편, 노르에피네프린 투여 여부와 같이 사건 수가 적은 변수는 회귀계수 추정이 불안정할 수 있으므로, 그 안정성을 Firth 로지스틱 회귀분석 (Firth's penalized logistic regression)으로 확인하였다 (Supplementary 2).

본 연구에서 소수 집단에 해당하는 치료 실패군의 사례를 인위적으로 늘리는 synthetic minority over-sampling technique (SMOTE) 기법은 결과 발생률을 왜곡하여 예측확률을 과대 추정 시키고 보정 성능을 악화시킬 우려가 있다[30]. 따라서 본 연구의 일차 분석에는 해당 클래스 불균형 (class imbalance) 보정 기법이 적절하지 않다고 판단하여 적용하지 않았다. 다만, 그 영향을 확인하기 위해 학습 자료에 한정하여 SMOTE를 적용한 민감도 분석을 별도로 수행하였다. 한편 추적 시점의 측정 시각이 환자마다 달라 이질성이 존재할 수 있으므로, 추적 측정시간 (HFNC 적용 시각부터 추적 활력징후 측정 시각까지의 경과 시간)의 중앙값과 사분위수 범위를 산출하고 치료 성공군과 실패군 간 차이를 Mann-Whitney U 검정으로 비교하였다. 아울러 측정 시점의 이질성이 결과에 미치는 영향을 확인하기 위하여, 추적 측정시간을 표준화한 값을 공변량으로 추가 보정한 모형과 추적 측정시간이 HFNC 적용 후 2시간 이내인 대상자로 제한한 모형을 민감도 분석으로 함께 적합하였다 (Supplementary 3).

#### 4) 모형 성능 및 변수 중요도

개발한 모형의 성능은 식별(discrimination), 보정(calibration), 분류(classification)의 세 측면에서 평가하였다. 식별 성능은 AUROC와 그 95% CI (DeLong 검정)으로 평가하였으며, 일차 다섯 개 변수 LR 모형과 단일 산소화 지표 모형 (추적 시점 S/F ratio, 추적 시점 ROX-심박수 지수) 간 식별 성능 차이는 DeLong 검정[31]으로 비교하였다 (Supplementary 4). 보정 성능은 Brier 점수, 보정 기울기 (calibration slope), 보정 절편 (calibration intercept), 그리고 국소 회귀 평활 (locally estimated scatterplot smoothing)을 적용한 보정 도표 (calibration plot)로 평가하였다[22,28]. 분류 성능은 임상 적용을 고려하여 Youden 지수 기반의 최적 임계값을 기준으로 민감도, 특이도, 양성 및 음성 예측도, 양성 및 음성 우도비를 산출하였다.

변수의 상대적 기여도는 사전 정의한 다섯 개 변수에 대해 상호 보완적인 세 가지 방법으로 평가하였다. RF에서는 Gini 불순도 감소량과 30회 반복 평균 순열 중요도 (permutation importance)를 산출하였고[32], GB에서는 Shapley additive explanations (SHAP) 평균 절댓값을 산출하였다[33].

### 6. 윤리적 고려

본 연구는 헬싱키 선언의 원칙에 따라 수행되었으며, 해당 의료기관 임상연구심의위원회의 승인을 받았다 (IRB Approval No. HKS 2025-04-015-005). 후향적 비식별 자료의 특성상 서면 동의 면제 승인을 받아 수행하였다.

## 연구 결과

### 1. 대상자의 일반적 특성

최종 분석 대상자 201명 중 33명 (16.4%)이 HFNC 치료 실패를 경험하였다. 대상자의 연령 중앙값은 79세 (IQR 70~86)였으며, 남성은 107명 (53.2%)이었다. 예측모형에 포함된 주요 변수의 분포를 살펴보면, 응급실 체류 중 노르에피네프린을 투여 받은 환자는 9명 (4.5%)이었고, 내원 시 기저 SOFA 총점 중앙값은 2점 (IQR 1~4), 기저 동맥혈 pH 중앙값은 7.36 (IQR 7.28~7.42)이었다. 대상자의 구체적인 일반적 특성은 Table 1과 같다.

### 2. 다변량 예측모형의 성능

**Figure 1.**
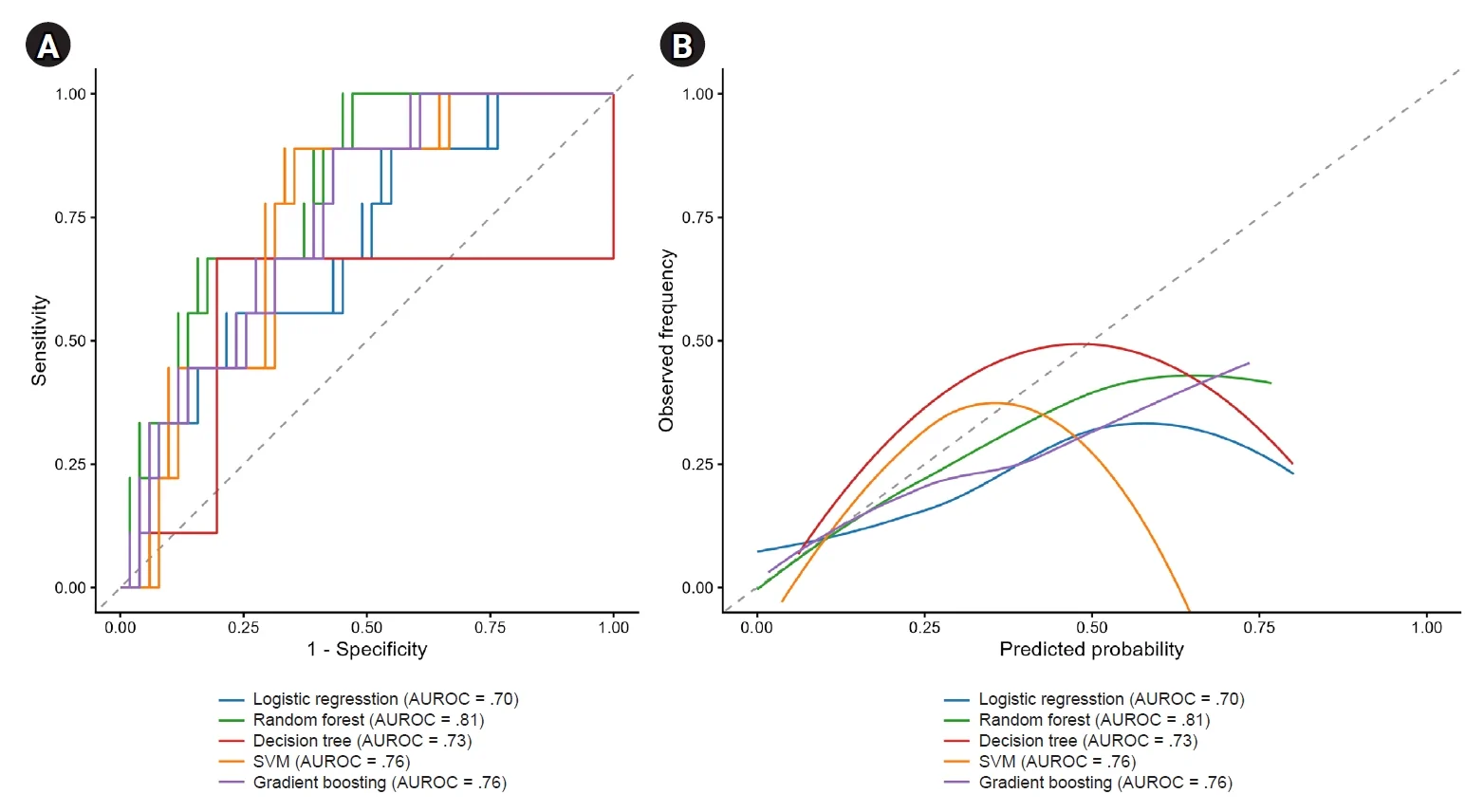
ROC curves (Figure 1-A) and calibration plots (Figure 1-B) on the held-out test set (n = 60). ROC = Receiver operating characteristic; AUROC = Area under the receiver operating characteristic curve; SVM = Support vector machine.

HFNC 치료 실패 예측을 위해 사전 정의한 다섯 개 변수를 이용하여 LR, RF, DT, SVM, GB 모형을 개발하고 30% 검증 자료 (n = 60)에서 성능을 비교하였다 (Table 2, Figure 1). 모형 성능 비교 결과, 다섯 모형의 식별 성능은 AUROC .70~.81 범위였다. RF가 .81 (95% CI .68–.95)로 가장 높았고, GB (.76, 95% CI .61–.91), SVM (.76, 95% CI .61–.91), DT (.73, 95% CI .55–.90)가 뒤를 이었으며, LR이 .70 (95% CI .51–.88)으로 가장 낮았다. 다섯 모형의 신뢰구간은 서로 중첩되었다.

보정 성능 지표인 보정 기울기와 보정 절편을 살펴본 결과, SVM (0.93, −0.20)과 RF (0.80, −0.48)가 이상적인 기준 (calibration slope 1, calibration intercept 0)에 가장 근접하였다. 반면 LR은 보정 기울기 0.38, 보정 절편 −1.00으로 기준에서 가장 크게 벗어났으며, DT (calibration slope 0.55)가 그 뒤를 이었다. Brier 점수는 RF 모형이 .112로 가장 낮았다. 임상 적용 시의 분류 성능의 경우, Youden 지수 기반 최적 임계값 (.073)에서 LR이 민감도 88.9%, 특이도 47.1%, 양성 예측도 22.9%, 음성 예측도 96.0%, 양성 우도비 1.68, 음성 우도비 0.24를 보였다.

### 3. 내적 검증 및 민감도 분석

LR의 내적 타당도 검증에서, 전체 자료에 대한 부트스트랩 낙관도 보정 결과 apparent AUROC는 .82, 낙관도는 .04였으며, 이를 반영한 낙관도 보정 AUROC (optimism-corrected AUROC)는 .78 (95% CI .70–.86)이었다. 무작위 시드 (random seed)를 변경한 층화 70:30 분할 50회 반복에서 평균 AUROC는 .77 (SD .07; 2.5~97.5 percentile .64~.93)이었다.

DeLong 검정에서 다섯 개 변수 LR (AUROC .70)은 단일 추적 시점 S/F ratio 모형 (AUROC .79; *p* = .185) 및 단일 추적 시점 ROX-심박수 지수 모형 (AUROC .77; *p* = .491)과 통계적으로 유의한 차이를 보이지 않았다 (Supplementary 4). 반복 10-겹 교차검증에서 다섯 개 변수 모형과 단일 추적 시점 S/F 모형의 AUROC는 각각 .78과 .77이었고 (ΔAUROC .017, 95% CI −.04–.08, *p* = .545), AUPRC는 .44와 .43, Brier 점수는 .114와 .117이었다. 보정 성능은 두 모형이 유사한 수준이었다 (calibration slope 0.68 vs. 0.87, calibration intercept −0.39 vs. −0.16). DCA에서 두 모형의 순이익 곡선은 대부분의 임계확률 구간에서 서로 근접하였다 (Supplementary 2, Supplementary Figure 1).

핵심 세 변수 (추적 시점 S/F ratio, 동맥혈 pH, SOFA 총점)로 구성한 간명 모형의 AUROC는 .73 (95% CI .56~.91)이었으며, 보정 기울기는 0.45, 보정 절편은 −0.88이었다. 학습 자료에 한정하여 SMOTE를 적용한 민감도 분석에서는 다섯 모형의 식별 성능은 AUROC .61~.78이었으며, 보정 기울기는 −0.06~0.76, Brier 점수는 .158~.237이었다.

LR의 회귀계수 분석에서 추적 시점 S/F ratio의 오즈비 (odds ratio, OR)는 0.20 (95% CI 0.07–0.58, *p* = .003), 동맥혈 pH는 0.55 (0.35–0.86, *p* = .009)로 유의한 예측변수로 확인되었으며, 노르에피네프린 투여 (OR 2.70, 95% CI 0.37–19.50, *p* = .325), 추적 시점 ROX-심박수 지수 (OR 0.85, *p* = .663), SOFA 총점 (OR 1.29, *p* = .290)은 통계적으로 유의하지 않았다. 노르에피네프린 오즈비의 CI가 넓어 Firth 로지스틱 회귀분석으로 추정 안정성을 확인하였다. 그 결과 노르에피네프린 투여의 오즈비는 2.58 (95% CI 0.37–16.68, *p* = .326)이었으며, 추적 시점 S/F ratio (OR 0.23, 95% CI 0.07–0.58, *p* < .001)와 동맥혈 pH (OR 0.57, 95% CI 0.36–0.86, *p* = .007)는 유의하였다 (Supplementary 2 Part A). 최종 로지스틱 회귀모형의 결과를 이용하여 개별 환자의 예측확률을 산출하면 다음과 같다. z = (측정값 − 평균) / 표준편차이며, 표준화에 사용한 평균과 표준편차는 Table 3에 제시하였다.


$$\log\mathrm{it}\;(p)=-2.5563\;-\;1.6230\;\times \;z\;(\mathrm{follow}-\mathrm{up}\;S/F\;\mathrm{ratio})\;-\;0.1577\;\times \;z\;(\mathrm{follow}-\mathrm{up}\;\mathrm{ROX}\;-\;\mathrm{HR}\;\mathrm{index})\;+\;0.9937\;\times \;\mathrm{norepinephrine}\;\mathrm{use}\;+\;0.2505\;\times \;z\;(\mathrm{SOFA}\;\mathrm{score})\;-\;0.5929\;\times \;z\;(\mathrm{arterial}\;\mathrm{pH})\;p\;=\;\frac{1}{1\;+\;e^{-\mathrm{logit}}}$$

### 4. 변수 중요도

사전 정의한 다섯 개 변수의 상대적 기여도를 RF의 Gini 불순도 감소량, 30회 반복 순열 중요도, GB의 SHAP 평균 절댓값으로 사후 분석하였다 (Table 4, Supplementary Figure 2). 분석 결과, 동맥혈 pH와 추적 시점 S/F ratio가 모든 평가 지표에서 일관되게 가장 강력한 예측 변수로 확인되었다. 구체적으로 RF의 Gini 불순도 감소량에서는 동맥혈 pH (100.0)가 가장 기여도가 높았고, 추적 시점 S/F ratio (79.1), 추적 시점 ROX-심박수 지수 (73.9), SOFA 총점 (58.3) 순이었다. 이러한 경향은 순열 중요도와 GB의 SHAP 분석에서도 유사하게 나타나, 동맥혈 pH와 추적 시점 S/F ratio가 상위권을 유지하였다.

반면, 노르에피네프린 투여 여부는 예측변수로서의 기여도가 뚜렷하게 낮았다. RF에서 분기 기준 변수로 선택되지 않아 Gini 불순도 감소량이 0.0이었으며, 순열 중요도와 SHAP 분석에서도 기여도가 모두 0으로 산출되어 세 지표 모두에서 최하위를 보였다 (Supplementary Figure 2).

## 논의

본 연구는 응급실 AHF 환자를 대상으로 산소화 반응, 혈역학적 불안정, 다장기 중증도, 산-염기 상태를 통합한 다섯 개 변수 예측모형을 개발하고 내적으로 검증하였다. 분석 결과, 낙관도 보정 후 AUROC는 .78 (95% CI .70~.86)이었으며, 추적 시점 S/F ratio와 동맥혈 pH가 HFNC 치료 실패의 예측변수로 확인되었다.

본 연구에서 구축한 네 가지 머신러닝 모형 (RF, DT, SVM, GB)은 LR에 비해 뚜렷한 식별 성능의 이점을 보이지 않았다. 이는 임상 예측모형에서 머신러닝이 LR보다 항상 우수하지는 않다는 체계적 문헌고찰[34] 결과와 일치한다. 머신러닝은 변수 간의 복잡한 비선형 관계가 존재하는 대규모 데이터에서 강점을 보이지만[35], 본 연구와 같이 임상적 타당성을 근거로 소수의 핵심 변수만을 선별한 조건에서는 LR만으로도 충분히 안정적인 예측력을 확보할 수 있었다. 특히 단일 의사결정나무 (DT)는 본 연구와 같이 사건 수가 제한적인 클래스 불균형 (class imbalance) 자료에서 예측확률이 소수의 값으로만 산출되어 민감도가 .67로 가장 낮게 나타나는 단일 트리 기반 모형의 한계를 보였다[17]. 한편 응급실에서 예측모형을 활용하려면 정확도뿐 아니라 결과의 해석 가능성이 중요하다[36]. 복잡한 앙상블 모형은 판단 과정이 불투명한 블랙박스 (black-box) 특성을 지니는 반면, LR은 개별 변수의 기여도를 오즈비 (odds ratio)로 직관적으로 제시하여 임상 적용과 의사소통에 유리하다[34,35]. 본 연구에서도 이러한 해석 가능성을 보완하기 위해 머신러닝 모형에 SHAP 기반 변수 기여도 분석을 함께 제시하였다. 다만 머신러닝이 더 큰 표본과 복잡한 변수 구조에서 성능 이점을 보일 가능성은 배제할 수 없으므로[35], 향후 대규모 다기관 코호트를 통한 외부 검증에서 두 접근의 성능을 재확인할 필요가 있다.

반복 교차검증 결과, 다변수 통합 모형과 단일 추적 시점 S/F 지표 모형 간의 식별 성능에는 유의한 차이가 없었다(ΔAUROC .017, 95% CI −.043~.078, *p* = .545). 이는 데이터 내 발생 사건(event)이 적을 때는 예측 변수를 추가하더라도 반드시 성능 향상으로 이어지지 않음을 보여준다. 즉, 단순히 변수를 늘리기보다 임상적 타당성을 갖춘 소수의 핵심 지표를 선별하여 적용하는 것이 더 효율적일 수 있다[28,29]. 특히, 본 연구에서는 단일 지표 모형이 다변수 모형과 유사한 수준의 식별 성능을 보였으며, 이는 촌각을 다투는 응급실 현장에서 간명한 선별 도구가 유용하게 쓰일 수 있음을 시사한다. 다만 연구에 포함된 사건이 33건에 그쳐, 두 모형 간의 미세한 성능 차이를 통계적으로 확증하기에는 한계가 있었다. 따라서 향후 대규모 표본을 확보하여, 다변수 모형을 적용했을 때 예측 성능이 유의미하게 높아지는지 재검증하는 후속 연구가 필요하다.

일반적으로 임상 예측 모형을 평가할 때는 환자를 잘 분류해 내는지를 확인하기 위해 식별 성능뿐만 아니라, 예측된 확률이 실제 발생률과 얼마나 일치하는지를 확인하기 위한 보정 성능을 함께 확인하도록 권장된다[22,28]. 본 연구의 민감도 분석에서 SMOTE를 적용하였을 때 식별 성능은 향상되지 않았고 보정 성능은 오히려 떨어졌다. 이는 인위적인 데이터 과대표집이 예측 확률의 분포를 왜곡하여 보정력을 악화시킬 수 있다는 van den Goorbergh 등[30]의 연구 결과와 맥을 같이 한다. 한편, 핵심 변수 3개만으로 구성한 간명 모형은 5개 변수 모형과 비슷한 수준의 식별 및 보정 성능을 보였다. 이는 발생 사건이 적은 상황에서 변수를 줄이더라도 모형의 성능이 충분히 유지됨을 뜻한다. 다만, 두 모형 모두 보정 기울기가 1에 미치지 못했고 검증 데이터의 사건 수가 적어 신뢰구간이 넓게 추정되었다는 한계가 있다. 따라서 현재 모형에서 산출된 위험도 수치를 임상에 곧바로 적용하는 것은 주의가 필요하며[22], 향후 외부 타당도 검증을 통해 간명 모형의 실질적인 효용성을 재확인하는 연구가 필요하다.

변수 중요도 분석결과, 추적 시점 S/F ratio와 동맥혈 pH가 HFNC 치료 실패의 핵심 예측지표로 확인되었다. 이는 HFNC의 치료 성패가 초기 산소화 반응뿐 아니라 환기·산–염기 상태와 함께 결정됨을 시사한다. 먼저, 동맥혈 pH는 Gini 불순도 감소량과 순열 중요도에서 가장 큰 기여를 보였다. 이는 AHF 환자의 전신적 악화를 포착하는 핵심 지표로, AHF에서 pH의 저하는 심박출량 감소에 따른 조직 저관류나, 폐부종 악화 및 호흡근 피로로 인한 고탄산혈증성 환기 부전이 진행되고 있음을 의미한다[3,19]. HFNC는 사강 제거 (dead-space washout)를 통해 경미한 고탄산혈증의 개선에는 도움을 줄 수 있으나, 본질적으로는 산소화를 지원하는 보존적 요법이므로 심각한 환기 부전이나 대사성 산증을 역전시키는 데에는 명확한 한계가 있다[9]. 따라서 내원 시 측정된 동맥혈 pH가 낮다는 것은 HFNC의 단순한 산소 투여만으로는 극복하기 어려운 중증 상태를 반영하며, 보다 침습적인 호흡 보조가 조기에 필요할 수 있음을 시사한다.

다음으로 주요 예측 변수로 확인된 추적 시점의 S/F ratio는 HFNC 적용 직후의 산소화 반응이 환자의 예후와 직결됨을 보여준다. 이는 치료 시작 후의 초기 산소화 반응이 기저 상태보다 치료 성패를 더 명확하게 반영한다는 다수의 성인 및 소아 호흡부전 선행 연구[15,16,24,37,38] 결과와 일치하는 결과이며, 산소화 상태를 나타내는 S/F ratio가 환기 및 산-염기 균형을 반영하는 동맥혈 pH와 상호 보완적으로 작용하여 두 지표를 함께 고려할 때 예측력이 향상된다는 기존 연구[3,19]와도 유사한 결과이다. 임상적으로 추적 시점의 S/F ratio가 낮게 유지된다는 것은 HFNC에 대한 생리적 산소화 반응이 불충분함을 뜻하며[15,16], 이는 조기에 호흡 보조 단계를 높여야 할 고위험 환자를 가려내는 중요한 임상적 신호가 된다[13,14]. 무엇보다 S/F ratio는 침습적인 동맥혈가스 분석 없이도 환자 곁에서 SpO_2_와 FiO_2_만으로 즉각 산출할 수 있어 바쁜 응급실 실무 환경에 매우 적합하다[39]. 따라서 간호사는 S/F ratio를 활용하여 산소화 반응을 비침습적으로 반복 사정함으로써 치료 실패 고위험군을 조기에 선별하고, 재사정 우선순위를 효율적으로 설정할 수 있다[7,8]. 다만, S/F ratio는 SpO_2_가 정상 범주로 높은 구간에서는 산소화 변화에 대한 민감도가 떨어지고 환기 상태를 직접 반영하지 못한다는 한계가 있으므로, 환자의 호흡 노력(work of breathing) 등 다른 임상 징후를 반드시 함께 종합하여 해석해야 한다.

반면, 노르에피네프린 투여의 경우 예측 시점 이전 투여로 정의를 제한한 결과 독립적 예측변수로 확인되지 않았으며, Firth 로지스틱 회귀분석에서도 동일하였고 변수 중요도 세 지표에서 모두 최하위였다. 이는 승압제 사용이 HFNC 실패에 선행하는 예측인자라기보다, 삽관 이후의 진정 및 양압환기에 뒤따르는 혈역학적 변화를 상당 부분 반영하고 있었을 가능성을 시사한다. 다만, 본 연구에서 예측 시점 이전에 승압제를 투여받은 환자가 9명에 그쳐 통계적 검정력이 제한적이었던 만큼, 혈역학적 불안정과 HFNC 실패 간의 연관성을 명확히 파악하기 위해서는 대규모 표본을 통한 후속 연구가 요구된다. 비록 본 예측 모형에서 주요 변수로 채택되지는 않았으나 승압제 투여가 필요한 상태는 그 자체로 환자의 높은 악화 위험을 뜻하는 핵심 임상 지표임을 고려하여, 응급실 간호사는 혈압, 말초 관류, 의식 수준 등 순환 부전 징후를 면밀히 모니터링하고, 이상 발생 시 의료진과 즉각적으로 소통하여 선제적 중재가 이루어지도록 해야 한다[7,8].

또한, 다변수 모형 분석 결과, 추적 시점의 ROX-심박수 지수와 SOFA 총점은 독립적인 예측 변수로 확인되지 않았다. 이들 변수는 선행연구를 근거로 사전 선정되었고 변수 간 다중공선성도 관찰되지 않았으나 최종적으로 유의성에 도달하지 못하였다. 이는 모형에 포함된 추적 시점 S/F ratio와 동맥혈 pH가 HFNC 실패의 두 핵심 병태생리인 초기 산소화 반응 및 환기·산-염기 상태를 이미 강력하게 대변했기 때문으로 해석된다[3]. 구체적으로, ROX-심박수 지수는 S/F ratio와 함께 산소화 상태를 반영하는 지표이나[15,16], 본 코호트에서는 S/F ratio의 예측 연관성이 상대적으로 더 강하게 작용하였다. 포괄적 중증도 지표인 SOFA 총점 역시 여러 장기의 기능을 합산하는 특성상 특정 병태를 세밀하게 지시하지 못하므로[26], 산소화 및 환기 지표가 이미 통제된 조건에서는 독립적인 기여도가 나타나지 않은 것으로 생각된다. 또한 선행연구에서 ROX 계열 지표의 유용성이 지속적으로 보고된 바 있으므로[15,16], 향후에는 단일 측정치를 넘어 지표의 변화 추세(trend)를 반영한 추가 분석이 이루어질 필요가 있다.

본 연구의 간호학적 의의는 예측모형이 간호사의 전문적 판단을 지원하는 보조 도구로 기능할 수 있음을 보여준 데 있다. 모형에 포함된 예측변수가 모두 응급실에서 일상적으로 측정 가능한 지표라는 점은 실무 적용 가능성을 뒷받침한다. 간호사는 HFNC 적용 후 추적 시점 S/F ratio를 반복적으로 사정하고 동맥혈가스 검사 결과를 함께 확인함으로써 환자의 악화 징후를 조기에 인지하고, 제한된 간호 자원을 치료 실패 고위험군에 우선 배분하며, 이상 징후 발생 시 의료진에게 적시에 보고하는 간호 주도적 중재 (nurse-led intervention)를 수행할 수 있다[7,8]. 다만 모형의 구체적 임상 이점은 단일기관 후향적 자료에 기반한 만큼, 향후 다기관 전향적 연구를 통한 외부 검증에서 확인될 필요가 있다.

본 연구는 다음과 같은 제한점을 가진다. 첫째, 단일기관의 후향적 자료에 기반한 내적 검증 연구이므로 결과를 일반화하는 데 한계가 있으며, 다기관 전향적 자료를 이용한 외부 검증이 필요하다[18]. 둘째, 노르에피네프린 투여는 예측 시점 이전에 개시된 경우로 정의를 제한하였으나, 투여 개시 시각이 분 단위로 구조화되어 기록되지 않아 예측 시점과 근접한 사례의 선후 판정에는 불확실성이 남는다. 또한 이 정의에 해당하는 대상자가 9명 (4.5%)에 그쳐 해당 변수의 효과를 추정할 검정력이 제한적이었다. 셋째, 전체 사건 수와 검증 자료의 실패 사례가 제한적이어서 성능 추정치의 신뢰구간이 넓게 나타났다. 특히 노르에피네프린의 오즈비는 신뢰구간이 0.37~19.50으로 매우 넓어, 연관성의 방향과 크기를 모두 확정할 수 없었다. 넷째, 좌심실 박출률과 임상 표현형 등 AHF의 이질성을 반영하는 변수가 포함되지 못하였고 추적 측정 시점도 일정하지 않았으므로, 이러한 변수를 구조화하여 수집하는 전향적 연구를 통해 모형을 보완할 필요가 있다[1]. 아울러 보조호흡근 사용, 호흡 노력과 같이 응급실 간호사가 침상에서 직접 관찰·사정할 수 있는 지표와 흉부 X선상 폐울혈 소견[40,41]을 통합한 예측모형은 간호 실무 적용성과 환자 악화의 조기 인지 능력을 한층 높일 수 있을 것이다.

## 결론

본 연구는 응급실 급성 심부전 환자를 대상으로 산소화 반응과 환기·산–염기 상태, 전신 중증도를 통합한 HFNC 치료 실패 예측모형을 개발하고 내적으로 검증하였다. 추적 시점 S/F ratio와 동맥혈 pH가 핵심 예측변수로 확인되어, HFNC 적용 후 초기 산소화 반응과 환기·산–염기 상태를 함께 평가하는 것이 치료 실패 위험을 조기에 선별하는 데 유용할 가능성을 시사한다. 응급실 간호사는 HFNC 적용 후 이러한 지표들을 통합적으로 사정함으로써 치료 실패 고위험 환자를 조기에 인지하고 적시에 의료진에게 보고하는 임상적 판단을 지원하는 보조 도구로 본 모형을 활용할 수 있을 것이다. 다만 본 연구는 단일기관 후향적 자료에 기반한 내적 검증 연구로, 다변량 모형은 단일 추적 시점 S/F ratio 모형 대비 통계적으로 유의한 식별 성능 향상을 보이지 않았으므로 그 부가적 가치는 잠정적으로 해석되어야 한다. 향후 다기관 전향적 연구를 통해 충분한 사건수를 확보하여 모형의 외부 타당도를 검증하고, 응급실 임상 현장에서 본 예측모형을 실제 적용하여 그 정확성과 유용성을 확인하는 실증적 연구를 제언한다.

## Figures and Tables

**Table 1.** Baseline Characteristics of the Study Participants (N = 201)

Characteristics	n (%) or Median [IQR]
Demographics and clinical status	
Age (years)	79 [70~86]
Men	107 (53.2)
Glasgow Coma Scale	15 [15~15]
Vital signs at emergency department arrival	
Systolic BP (mmHg)	154 [134~171]
Diastolic BP (mmHg)	89 [77~103]
Heart rate (/min)	110 [93~124]
Respiratory rate (/min)	25 [20~29]
Body temperature (°C)	36.5 [36.0~37.0]
SpO_2_ (%)	96 [91~99]
Mean arterial pressure (mmHg)	112 [96~125]
Vasopressor use	
Norepinephrine	9 (4.5)
Laboratory findings	
Platelet count (×10^3^/μL)	216 [157~271]
Albumin (g/dL)	3.9 [3.5~4.2]
Total bilirubin (mg/dL)	0.70 [0.50~1.10]
Creatinine (mg/dL)	1.25 [0.94~1.73]
BNP (pg/mL)	1,106 [575~1,932]
Arterial blood gas	
pH	7.36 [7.28~7.42]
PaCO_2_ (mmHg)	40.5 [32.9~50.0]
PaO_2_ (mmHg)	68 [55~88]
Lactate (mmol/L)	2.0 [1.3~3.3]
HCO_3_^-^ (mEq/L)	22.6 [19.2~26.0]
Oxygenation indices at baseline	
ROX index	11.76 [4.70~17.14]
ROX-HR index	9.84 [4.67~16.78]
S/F ratio	293 [111~455]
SOFA score	2 [1~4]

IQR = Interquartile range; BP = Blood pressure; SpO_2_ = Peripheral oxygen saturation; BNP = B-type natriuretic peptide; PaCO_2_ = Arterial partial pressure of carbon dioxide; PaO_2_ = Arterial partial pressure of oxygen; ROX = Ratio of SpO_2_/FiO_2_ to respiratory rate; ROX-HR = Ratio of SpO_2_/FiO_2_ to respiratory rate × heart rate; S/F = SpO_2_/FiO_2_; SOFA = Sequential Organ Failure Assessment.

**Table 2.** Predictive Performance of the Multivariable Models

Model	AUROC (95% CI)	Calibration slope	Calibration intercept	Brier score	Threshold	Sensitivity	Specificity	PPV	NPV	LR+	LR−
Logistic regression	.70 (.51–.88)	0.38	−1.00	.139	.073	.89	.47	.23	.96	1.68	0.24
Random forest	.81 (.68–.95)	0.80	−0.48	.112	.092	1.00	.55	.28	1.00	2.22	0.00
Decision tree	.73 (.55–.90)	0.55	−0.74	.130	.182	.67	.80	.38	.93	3.40	0.42
SVM	.76 (.61–.91)	0.93	−0.20	.121	.160	.89	.67	.32	.97	2.67	0.17
Gradient boosting	.76 (.61–.91)	0.68	−0.66	.121	.134	.89	.57	.27	.97	2.06	0.20

Models were evaluated on the held-out test set (n = 60). Sensitivity, specificity, PPV, NPV, and likelihood ratios were computed at the Youden-optimal threshold. AUROC = Area under the receiver operating characteristic curve; CI = Confidence interval; PPV = Positive predictive value; NPV = Negative predictive value; LR+ = Positive likelihood ratio; LR− = Negative likelihood ratio; SVM = Support vector machine.

**Table 3.** Final Logistic Regression Model for High-flow Nasal Cannula Treatment Failure

Predictor	β	SE	OR	95% CI	*p*
Intercept	−2.56	0.39	0.08	0.04–0.17	< .001
Follow-up S/F ratio (standardized)	−1.62	0.55	0.20	0.07–0.58	.003
Follow-up ROX-HR index (standardized)	−0.16	0.36	0.85	0.42–1.74	.663
Norepinephrine use (0 = no, 1 = yes)	0.99	1.01	2.70	0.37–19.50	.325
SOFA score (standardized)	0.25	0.24	1.29	0.81–2.04	.290
Arterial pH (standardized)	−0.59	0.23	0.55	0.35–0.86	.009

The model was developed on the full cohort (n = 201, 33 events). Continuous predictors were standardized to a mean of 0 and a standard deviation of 1; the values used were (mean ± SD): follow-up S/F ratio (154.946 ± 57.265), follow-up ROX-HR index (7.358 ± 4.053), SOFA score (2.920 ± 2.342), and arterial pH (7.348 ± 0.111). Norepinephrine use was coded as 0 (no) or 1 (yes). Confidence intervals are Wald intervals. z = (observed value − mean) / SD. SE = Standard error; OR = Odds ratio; CI = Confidence interval; S/F = SpO_2_/FiO_2_; ROX-HR = Ratio of SpO_2_/FiO_2_ to respiratory rate × heart rate; SOFA = Sequential Organ Failure Assessment.

**Table 4.** Variable Importance for HFNC Treatment Failure

Predictor	Random forest Gini importance	Rank	Permutation importance (AUROC decrease, mean of 30 reps)	Rank	Mean |SHAP|	Rank
pH	100.0	1	.076	1	0.69	2
Follow-up S/F ratio	79.1	2	.054	2	0.82	1
Follow-up ROX-HR index	73.9	3	.032	3	0.36	4
SOFA score	58.3	4	.026	4	0.43	3
Norepinephrine use	0.0	5	.000	5	0.00	5

HFNC = High-flow nasal cannula; AUROC = Area under the receiver operating characteristic curve; SHAP = Shapley additive explanations; S/F = SpO_2_/FiO_2_; ROX-HR = Ratio of SpO_2_/FiO_2_ to respiratory rate × heart rate; SOFA = Sequential Organ Failure Assessment.

## SUPPLEMENTARY MATERIALS

Supplementary 1. Candidate variables: measurement timing, LASSO bootstrap stability, and variance inflation factor

Supplementary 2. Firth's penalized logistic regression and repeated cross-validation

Supplementary 3. Sensitivity analysis adjusting for the elapsed time to follow-up measurement

Supplementary 4. Comparison of the five-variable model with single oxygenation-index models

Supplementary Figure 1. Decision curve analysis comparing the five-variable model and the follow-up S/F ratio model (20 repeats of 10-fold cross-validation).

Supplementary Figure 2. Variable importance analysis of the five pre-specified predictors (panel A: Gini impurity decrease from random forest; panel B: permutation importance; panel C: mean absolute SHAP value).
