# Supplementary material for: Development of a predictive model for high-flow nasal cannula treatment failure in emergency department patients with acute heart failure: a retrospective secondary data analysis
Source: J Korean Biol Nurs Sci. 2026 Aug 25;28(3):528–39. [Article in Korean] doi: 10.7586/jkbns.26.030 (PMC13539167; doi:10.7586/jkbns.26.030)
Supplement: Supplementary file 1 [file jkbns-26-030-Supplementary-1.pdf]

Supplementary 1. Candidate variables: measurement timing, LASSO bootstrap stability, and variance inflation factor

Part A. Measurement timing and LASSO bootstrap stability selection

Variable	Measurement timepoint, min (median [IQR])	Selection frequency	Stability
Age	Baseline	0.011	Low (<0.1)
Glasgow Coma Scale	Baseline	0.082	Low (<0.1)
Baseline systolic BP	Baseline	0.006	Low (<0.1)
Baseline diastolic BP	Baseline	0.004	Low (<0.1)
Baseline heart rate	Baseline	0.002	Low (<0.1)
Baseline respiratory rate	Baseline	0.012	Low (<0.1)
Baseline body temperature	Baseline	0.005	Low (<0.1)
Baseline SpO_2	Baseline	0.023	Low (<0.1)
Baseline mean arterial pressure	Baseline	0.005	Low (<0.1)
Norepinephrine use	Before intubation	0.010	Low (<0.1)
Platelet count	Baseline	0.174	Moderate (0.1–0.5)
Albumin	Baseline	0.011	Low (<0.1)
Total bilirubin (log)	Baseline	0.049	Low (<0.1)
Creatinine (log)	Baseline	0.020	Low (<0.1)
BNP (log)	Baseline	0.002	Low (<0.1)
Arterial pH	Baseline	0.179	Moderate (0.1–0.5)
Arterial $PaCO_2$	Baseline	0.573	Stably selected (≥0.50)
Arterial PaO_2	Baseline	0.038	Low (<0.1)
Lactate (log)	Baseline	0.006	Low (<0.1)
HCO_3^-	Baseline	0.017	Low (<0.1)
Baseline ROX index	Baseline	0.007	Low (<0.1)
Baseline ROX-HR index	Baseline	0.002	Low (<0.1)
Baseline S/F ratio	Baseline	0.027	Low (<0.1)
SOFA score	Baseline	0.766	Stably selected (≥0.50)
Follow-up systolic BP	68 [53–86]	0.052	Low (<0.1)
Follow-up diastolic BP	68 [53–86]	0.013	Low (<0.1)
Follow-up heart rate	68 [53–86]	0.276	Moderate (0.1–0.5)
Follow-up respiratory rate	68 [53–86]	0.033	Low (<0.1)
Follow-up body temperature	68 [53–86]	0.013	Low (<0.1)
Follow-up SpO_2	68 [53–86]	0.071	Low (<0.1)
Follow-up mean arterial pressure	68 [53–86]	0.008	Low (<0.1)
Follow-up ROX index	68 [53–86]	0.001	Low (<0.1)
Follow-up ROX-HR index	68 [53–86]	0.061	Low (<0.1)
Follow-up S/F ratio	68 [53–86]	0.385	Moderate (0.1–0.5)

Part B. Variance inflation factors (VIF) for the five pre-specified predictors

Predictor	VIF	Interpretation
Norepinephrine use	1.36	No multicollinearity
Follow-up S/F ratio	1.20	No multicollinearity
Follow-up ROX-HR index	1.18	No multicollinearity
SOFA score	1.35	No multicollinearity
pH	1.05	No multicollinearity

B = 1,000 half-sample bootstrap iterations with LASSO regression (λ1se); variables with a selection frequency ≥ 0.50 were considered stably selected. Log-transformed variables are denoted as (log).

BNP = B-type natriuretic peptide; BP= blood pressure; HCO_3^- = Bicarbonate; LASSO = Least absolute shrinkage and selection operator; $PaCO_2$ = Arterial partial pressure of carbon dioxide; PaO_2 = Arterial partial pressure of oxygen; ROX = Ratio of SpO_2/FiO_2 to respiratory rate; ROX-HR = Ratio of SpO_2/FiO_2 to respiratory rate × heart rate; S/F = SpO_2/FiO_2; SOFA = Sequential Organ Failure Assessment; SpO_2 = Peripheral oxygen saturation.

https://doi.org/10.7586/jkbns.26.030
