# Supplementary material for: Development of a predictive model for high-flow nasal cannula treatment failure in emergency department patients with acute heart failure: a retrospective secondary data analysis
Source: J Korean Biol Nurs Sci. 2026 Aug 25;28(3):528–39. [Article in Korean] doi: 10.7586/jkbns.26.030 (PMC13539167; doi:10.7586/jkbns.26.030)
Supplement: Supplementary file 2 [file jkbns-26-030-Supplementary-2.pdf]

Supplementary 2. Firth's penalized logistic regression and repeated cross-validation

Part A. Firth's penalized logistic regression for the five pre-specified predictors

Predictor	OR	95% CI	*p*
Norepinephrine use	2.58	0.37–16.68	.326
Follow-up S/F ratio	0.23	0.07–0.58	< .001
Follow-up ROX-HR index	0.89	0.42–1.69	.741
SOFA score	1.27	0.81–2.00	.304
pH	0.57	0.36–0.86	.007

Part B. Repeated 10-fold cross-validation: five-variable model versus follow-up S/F ratio model

Model	AUROC	AUPRC	Brier	Calibration slope	Calibration intercept
Five-variable model	.78	.44	.114	0.68	–0.39
Follow-up S/F ratio only	.77	.43	.117	0.87	–0.16

Part A: Firth's penalized logistic regression was fitted on the full cohort (N=201) with standardized continuous predictors.
CI = Confidence interval; OR = Odds ratio; ROX-HR = Ratio of SpO_2/FiO_2 to respiratory rate × heart rate; S/F = SpO_2/FiO_2; SOFA = Sequential Organ Failure Assessment.
Part B: Values are means of out-of-fold predictions from 20 repeats of stratified 10-fold cross-validation.
AUPRC = Area under the precision-recall curve; AUROC = Area under the receiver operating characteristic curve.
