# Supplementary material for: Development of a predictive model for high-flow nasal cannula treatment failure in emergency department patients with acute heart failure: a retrospective secondary data analysis
Source: J Korean Biol Nurs Sci. 2026 Aug 25;28(3):528–39. [Article in Korean] doi: 10.7586/jkbns.26.030 (PMC13539167; doi:10.7586/jkbns.26.030)
Supplement: Supplementary file 3 [file jkbns-26-030-Supplementary-3.pdf]

JKBNS

Supplementary 3. Sensitivity analysis adjusting for the elapsed time to follow-up measurement

Part A. Five-variable logistic regression without adjustment for elapsed time

Predictor	OR	95% CI	*p*
Norepinephrine use	2.70	0.37–19.50	.325
Follow-up S/F ratio	0.20	0.07–0.58	.003
Follow-up ROX-HR index	0.85	0.42–1.74	.663
SOFA score	1.29	0.81–2.04	.290
pH	0.55	0.35–0.86	.009

Part B. Five-variable logistic regression adjusted for elapsed time to follow-up measurement

Predictor	OR	95% CI	*p*
Norepinephrine use	2.83	0.39–20.60	.303
Follow-up S/F ratio	0.20	0.07–0.59	.004
Follow-up ROX-HR index	0.86	0.43–1.75	.681
SOFA score	1.27	0.80–2.02	.316
pH	0.54	0.34–0.85	.007
Elapsed time to follow-up measurement	0.86	0.57–1.30	.483

Part C. Five-variable logistic regression restricted to patients whose follow-up measurement was obtained within 2 hours after HFNC initiation (n=165, 28 events)

Predictor	OR	95% CI	*p*
Norepinephrine use	6.94	0.78–61.79	.082
Follow-up S/F ratio	0.23	0.08–0.72	.011
Follow-up ROX-HR index	0.54	0.22–1.32	.178
SOFA score	0.99	0.58–1.69	.974
pH	0.50	0.31–0.83	.007

Part A presents the original five-variable logistic regression (Table 4) without adjustment for elapsed time to follow-up measurement. Part B presents the same model with elapsed time to follow-up measurement (standardized) added as an additional covariate. The predictive effects of follow-up S/F ratio (OR 0.20→0.20, p = .003→.004) and arterial pH (OR 0.55→0.54, p = .009→.007) remained stable after adjustment, and elapsed time itself was not a significant predictor (p = .483), indicating that timing heterogeneity of follow-up measurements did not substantially affect the study results. Part C presents the same model restricted to the 165 patients (28 events) whose follow-up measurement was obtained within 2 hours after HFNC initiation; follow-up S/F ratio (OR 0.23, p = .011) and arterial pH (OR 0.50, p = .007) remained independent predictors, and the model AUROC was .83.

CI = Confidence interval; OR = Odds ratio; ROX-HR = Ratio of SpO_2/FiO_2 to respiratory rate × heart rate; S/F = SpO_2/FiO_2; SOFA = Sequential Organ Failure Assessment.
