# Supplementary material for: Development of a predictive model for high-flow nasal cannula treatment failure in emergency department patients with acute heart failure: a retrospective secondary data analysis
Source: J Korean Biol Nurs Sci. 2026 Aug 25;28(3):528–39. [Article in Korean] doi: 10.7586/jkbns.26.030 (PMC13539167; doi:10.7586/jkbns.26.030)
Supplement: Supplementary file 4 [file jkbns-26-030-Supplementary-4.pdf]

Supplementary 4. Comparison of the five-variable model with single oxygenation-index models

Model	AUROC (95% CI)	Brier	Cal. slope	Cal. intercept	DeLong p vs. LR
Five-variable logistic regression (reference)	.70 (.51–.88)	.139	0.38	−1.00	–
Follow-up S/F ratio only	.79 (.58–.99)	.106	0.53	−0.73	.185
Follow-up ROX-HR index only	.77 (.57–.96)	.116	2.67	2.33	.491

DeLong test p-values compare each single-index model against the five-variable logistic regression.
AUROC = Area under the receiver operating characteristic curve; Cal. = Calibration; CI = Confidence interval; LR = Logistic regression; ROX-HR = Ratio of SpO_2/FiO_2 to respiratory rate × heart rate; S/F = SpO_2/FiO_2.

https://doi.org/10.7586/jkbns.26.030
