# Supplementary material for: Development of a predictive model for high-flow nasal cannula treatment failure in emergency department patients with acute heart failure: a retrospective secondary data analysis
Source: J Korean Biol Nurs Sci. 2026 Aug 25;28(3):528–39. [Article in Korean] doi: 10.7586/jkbns.26.030 (PMC13539167; doi:10.7586/jkbns.26.030)
Supplement: Supplementary file 5 [file jkbns-26-030-Supplementary-Figure-1.pdf]

JKBNS

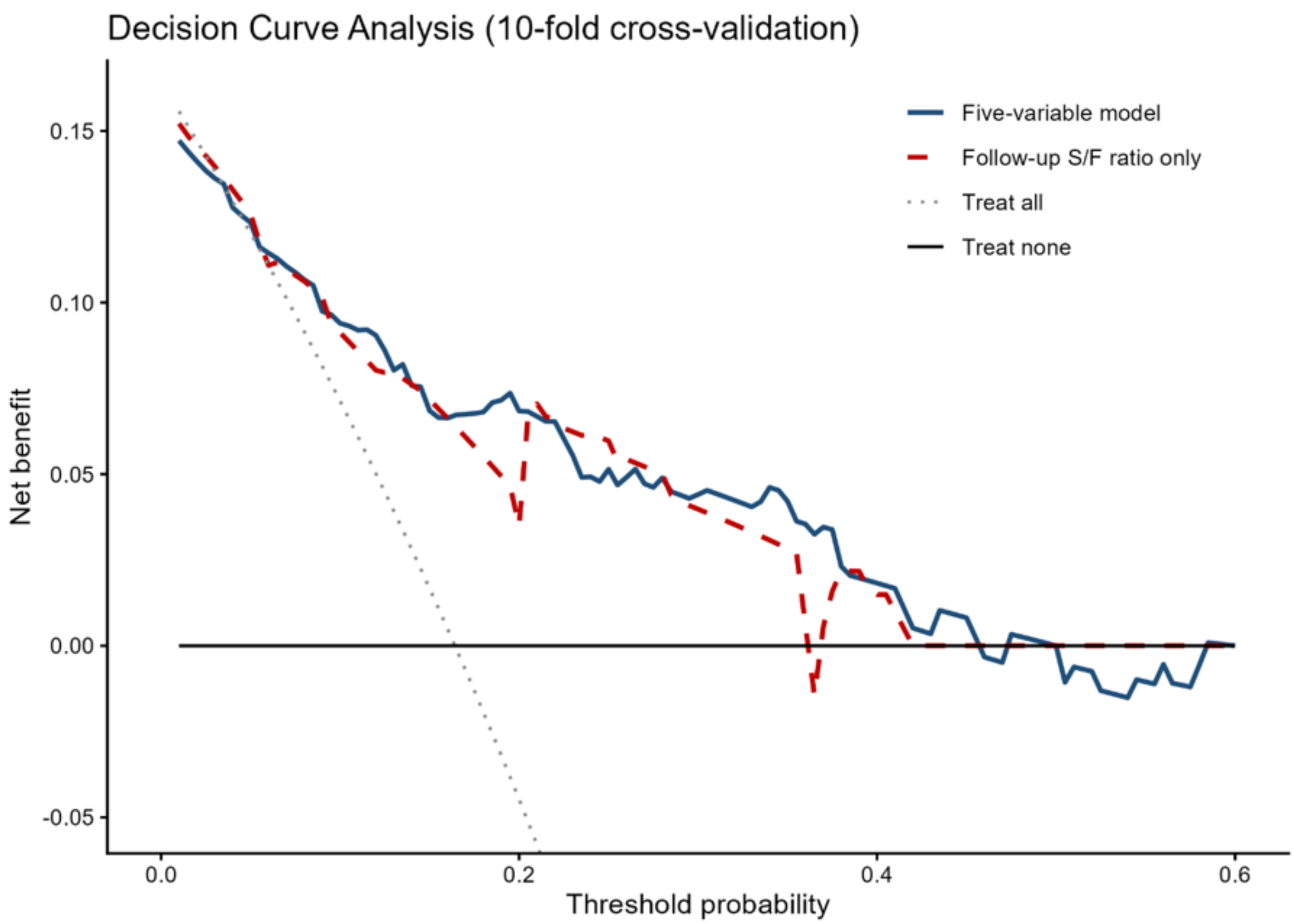

Supplementary Figure 1. Decision curve analysis comparing the five-variable model and the follow-up S/F ratio model (20 repeats of 10-fold cross-validation).
