# Supplementary material for: Development of a predictive model for high-flow nasal cannula treatment failure in emergency department patients with acute heart failure: a retrospective secondary data analysis
Source: J Korean Biol Nurs Sci. 2026 Aug 25;28(3):528–39. [Article in Korean] doi: 10.7586/jkbns.26.030 (PMC13539167; doi:10.7586/jkbns.26.030)
Supplement: Supplementary file 6 [file jkbns-26-030-Supplementary-Figure-2.pdf]

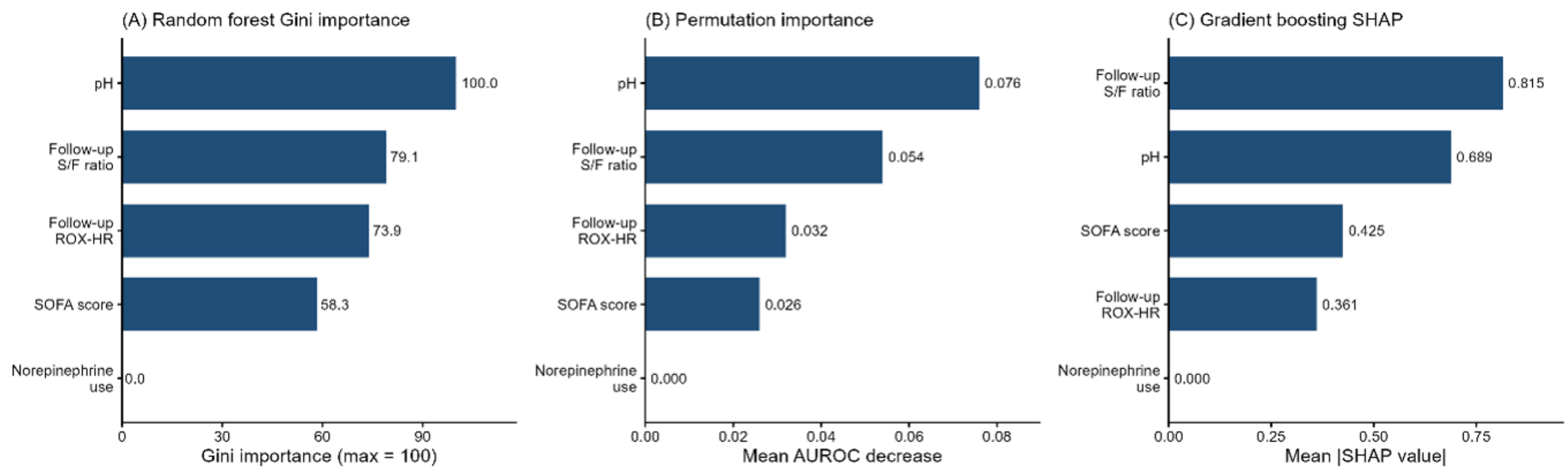

Supplementary Figure 2. Variable importance analysis of the five pre-specified predictors (panel A: Gini impurity decrease from random forest; panel B: permutation importance; panel C: mean absolute SHAP value).
